# Chemical Profiling and Comparison of Sangju Ganmao Tablet and Its Component Herbs Using Two-Dimensional Liquid Chromatography to Explore Compatibility Mechanism of Herbs

**DOI:** 10.3389/fphar.2018.01167

**Published:** 2018-10-16

**Authors:** Shuai Ji, Zhan-Zhong Liu, Jing Wu, Yan Du, Zhen-Yu Su, Tian-Yun Wang, Jie Han, Dong-Zhi Yang, Meng-Zhe Guo, Dao-Quan Tang

**Affiliations:** ^1^Department of Pharmaceutical Analysis, Xuzhou Medical University, Xuzhou, China; ^2^Jiangsu Key Laboratory of New Drug Research and Clinical Pharmacy, Xuzhou Medical University, Xuzhou, China; ^3^Department of Pharmacy, Xuzhou Infectious Disease Hospital, Xuzhou, China; ^4^Department of Pharmaceutical Analysis, Jiangsu College of Nursing, Huai’an, China

**Keywords:** two-dimensional liquid chromatography (2D-LC), hydrophilic interaction chromatography (HILIC), reverse-phase liquid chromatography (RPLC), Sangju Ganmao tablet (SGT), quadrupole time-of-flight mass spectrometry (qTOF-MS), compatibility mechanism

## Abstract

Sangju Ganmao tablet (SGT), a well-known Chinese patent medicine used to treat cold symptoms, is made from eight herbal medicines. In this study, an off-line hydrophilic interaction × reversed-phase two-dimensional liquid chromatography (HILIC × RP 2D-LC) method was developed to comprehensively separate the chemical constituents of SGT. Through optimization of the experimental conditions, a total of 465 peaks were finally detected in SGT, and the structures of 54 selected compounds were fully identified or tentatively characterized by quadrupole time-of-flight mass spectrometry (qTOF-MS) analysis. The established 2D-LC analysis showed high orthogonality (63.62%) and approximate 11-fold improvement in peak capacity (2399 and 1099, obtained by two calculation methods), in contrast to conventional one-dimensional RPLC separation. The eight component herbs of SGT were also respectively separated by using the 2D-LC system, and we found that a total of 12 peaks detected in SGT were not discovered in any component herbs. These newly generated chemical constituents would benefit better understanding of the compatibility mechanism of the component herbs. The strategy established in this study could be used for systematic chemical comparison of SGT and its component herbs, which contributes to exploration of herbal compatibility mechanism.

## Introduction

Most of the Chinese patent medicines are composed of several or even 10s of herbal medicines, and they are very complicated chemical systems containing both hydrophilic and hydrophobic compounds (Li et al., 2010). While profiling the chemical constituents is critically important for modern investigations of a Chinese patent medicine, chromatographic separation of such a complex system is still a big challenge. On the other hand, the chemical profiling of a Chinese patent medicine is usually different from its component herbs, since chemical reactions and physical changes, such as oxidation and precipitation, sometimes occur during decocting together (Kim et al., 2014). Investigating the different chemical constituents between a Chinese patent medicine and its component herbs may be one of effective approaches to explore its compatibility mechanism.

Sangju Ganmao tablet (SGT), a well-known Chinese patent medicine, is currently used in clinical practice to treat cold symptoms, and has been officially recorded in China Pharmacopoeia (Chinese Pharmacopoeia Commission, 2015). It is composed of eight herbs, namely mulberry leaf (leaves of *Morus alba* L.), chrysanthemum (flowers of *Chrysanthemum morifolium* Ramat.), Fructus *Forsythiae* [fruits of *Forsythia suspensa* (Thunb.) Vahl], licorice (roots and rhizomes of *Glycyrrhiza uralensis* Fisch.), Semen *Armeniacae* Amarum (seeds of *Prunus armeniaca* L.), *Platycodi* Radix [roots of *Platycodon grandiflorus* (Jacq.) A.DC.], *Phragmitis* Rhizoma [roots and rhizomes of *Phragmites australis* (Cav.) Trin. ex Steud.], and mint (whole herbs of *Mentha canadensis* L.). The chemical constituents of SGT mainly include flavonoids (free flavonoids and flavonoid glycosides), triterpenoid saponins, phenylethanoid glycosides, and organic acids (Chang et al., 2014). Although several researches have studied the chemical profiling of these eight component herbs using various methods (Huang and Sheu, 2007; He et al., 2013; Chen et al., 2016; Song et al., 2017), systematic chemical analysis of SGT has never been reported so far. Recently, we developed a LC–MS method to profile the chemical constituents of SGT by optimizing different HPLC systems, and only less than 50 compounds were detected (Guo et al., 2017). Comprehensive profiling of chemical constituents in SGT is hindered due to its complex chemical composition, and the significantly different content renders those minor components difficult to be separated and detected. The chemical differences of SGT and its component herbs are also unknown.

In the past decade, two-dimensional liquid chromatography (2D-LC) has been proven to be a powerful tool in rapid separation and detection of chemical constituents in traditional Chinese medicines (TCM) (Shellie and Haddad, 2006; Guiochon et al., 2008; Marchetti et al., 2008). Among all the separation modes, combination of reversed-phase liquid chromatography (RPLC) and hydrophilic interaction chromatography (HILIC) is an effective method to separate complex mixtures with a wide range of polarities (Wang et al., 2008; Jandera and Hájek, 2018). A 2D-LC system can be operated in the on-line or off-line mode. Comparing with an on-line 2D-LC system with complex equipment settings, an off-line 2D-LC system can easily accomplish the flexible integration of different separation mechanisms without instrumental limitation (Li et al., 2014). Very recently, an off-line HILIC × RP 2D-LC method was established in our lab to achieve the comprehensive profiling of the chemical constituents of *Ginkgo biloba* extract (Ji et al., 2017).

In this study, a method based on off-line HILIC × RP 2D-LC coupled with qTOF-MS was established to comprehensively profile the chemical constituents of SGT, and the 2D-LC system was systematically optimized to achieve ideal orthogonality and peak capacity. The established method was used to investigate the chemical differences between SGT and its component herbs in order to explore the herbal compatibility mechanism.

## Materials and Methods

### Chemicals, Reagents, and Materials

The reference standards of isorhmnetin (**3**), diosmetin (**6**), isochlorogenic acid A (**7**), kaempferol (**10**), quercetin (**14**), isochlorogenic acid C (**15**), apigenin (**19**), forsythin (**20**), chlorogenic acid (**23**), liquiritin (**25**), luteolin-7-*O*-glucoside (**26**), astragalin (**28**), luteolin (**29**), buddleoside (**31**), quercetin-3-*O*-glucoside (**33**), amygdalin (**37**), caffeic acid (**38**), forsythoside A (**39**), glycyrrhizic acid (**40**), ferulic acid (**42**), rutin (**44**), hyperoside (**50**), and platycodin D (**51**) were purchased from Sichuan Weikeqi Biological Technology, Co., Ltd. (Chengdu, China). Mulberry leaf, chrysanthemum, Fructus *Forsythiae*, licorice, Semen *Armeniacae* Amarum, *Platycodi* Radix and *Phragmitis* Rhizoma were purchased from Guangdong Huiqun Chinese Traditional Medicine, Co., Ltd. (Shantou, China), and mint was provided by Jiangxi Jianmin Natural Perfume Factory (Ji’an, China). Their voucher specimens have been deposited at the Department of Pharmaceutical Analysis, Xuzhou Medical University, Xuzhou, China. All other reagents were of analytical grade and obtained commercially.

### Sample Preparation

The simulative solution of SGT was prepared according to its record in China Pharmacopoeia (Chinese Pharmacopoeia Commission, 2015), and the detailed experimental process is described in Supporting information. A total of 23 reference standards were weighed accurately and dissolved in methanol to obtain their individual standard solutions at the concentration of 1 mg/mL, except for **26** (0.25 mg/mL). To optimize chromatographic condition of the 2D-LC system, the solutions of compounds **7**, **15**, **19**, **20**, **23**, **25**, **26**, **28**, **33**, **37, 38, 39, 40**, **42**, **44**, and **51** were mixed, dried under a gentle nitrogen flow, and reconstituted in methanol to produce mixed standard solutions. All the solutions were stored at 4 °C until use.

### Off-Line Two-Dimensional Liquid Chromatography

The following HILIC columns were studied: Agilent Zorbax HILIC plus (2.1 mm × 150 mm, 3.5 μm, Agilent, Santa Clara, CA, United States) and Waters XBridge Amide (2.1 mm × 150 mm, 3.5 μm, Waters, Milford, MA, United States). For RPLC, Agilent Zorbax SB-C8 (4.6 mm × 150 mm, 5.0 μm), Agilent Zorbax SB-C18 (2.1 mm × 100 mm, 3.5 μm), Agilent Eclipse plus C18 (2.1 mm × 100 mm, 3.5 μm) and Agilent Eclipse plus C18 (4.6 mm × 100 mm, 3.5 μm) columns were studied.

The D1 LC (HILIC) was conducted on a Waters e2695 HPLC system, and the samples were separated on a Waters XBridge Amide column (2.1 mm × 150 mm, 3.5 μm). The mobile phase consisted of 0.1% formic acid (A) and acetonitrile (B), and a gradient elution program was used: 0 min, 90% B; 10 min, 60% B. The flow rate was 0.4 mL/min, and the column temperature was 35°C. The detection wavelength was 254 nm. From 1 to 10 min, a total of 10 fractions (Fraction 1 to Fraction 10) were collected with a one-min interval. The sample injection was repeated for five times, and the same fractions were combined, respectively. The resulting fractions (except for Fraction 1) was concentrated to dryness under a gentle flow of nitrogen gas at 37°C, and were then re-dissolved in 100 μL of 50% methanol for the next D2 LC analysis.

The D2 LC (RPLC) was performed on an Agilent 1290 UHPLC system. The samples were separated on an Agilent Eclipse Plus C18 column (4.6 mm × 100 mm, 3.5 μm), and a binary mobile phase composed of 0.1% formic acid (A) and methanol (B) was used following a gradient elution program: 0 min, 5% B; 30 min, 95% B. The flow rate was 1.0 mL/min, and the column temperature was 35°C. The detection wavelength was 254 nm.

### Mass Spectrometry

An Agilent 6550 qTOF mass spectrometer equipped with an electrospray interface (ESI) was coupled to the D2 LC system, and the ion source was operated in the negative ion mode. The D2 LC eluent from the UV detector was introduced into the mass spectrometer by using a T-splitter, and the post-column splitting ratio was 8:1 (approximate 0.11 mL/min into the mass spectrometer). The MS parameters were set as follows: ionization mode, Dual AJS ESI (-); reference ion, 112.98 and 1033.98; capillary voltage, 3500 V; drying temperature, 280°C; dry gas flow, 12 L/min; atomizer pressure, 28 psig; sheath temperature, 350°C; sheath gas flow, 12 L/min; scan range, *m/z* 50–1500; cataclastic voltage, 300 V; collision energy, 15 and 45 eV.

### Data Analysis

The raw DAD data were exported into OriginPro 9.1 software (OriginLab Corporation, United States) to construct the contour plots. Orthogonality (*O*) and peak capacity (*n*_2D_) were calculated based on literatures (Gilar et al., 2005; Rutan et al., 2012), as described in our previous publication (Ji et al., 2017).

## Results and Discussion

### Optimization of the Off-Line 2D-LC System

**FIGURE 1:**
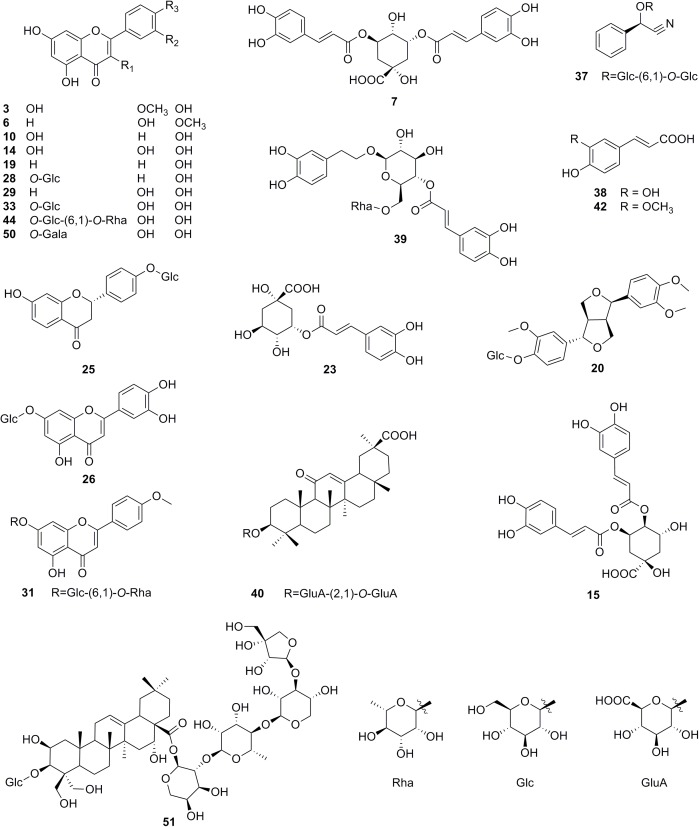
Chemical structures of 23 reference standards from SGT.

The separation modes of D1 and D2 in a 2D-LC system are determined based on the types of interactions between samples and stationary phases. The separation capacity of RPLC is mainly associated with non-polar selectivity, while HILIC is dominated by polar interaction. Therefore, the two different separation modes were combined to enhance orthogonality and peak capacity in this study, since the chemical constituents of SGT have a wide range of polarities. RPLC has been selected as the second dimension since it usually shows better peak shape of analytes and better compatibility with mass spectrometry, according to literatures (Jandera and Hájek, 2018). Among the 23 reference standards, 16 representative compounds (**7**, **15**, **19**, **20**, **23**, **25**, **26**, **28**, **33**, **37, 38, 39, 40**, **42**, **44,** and **51**), which are the major constituents of SGT with different structural types, were chosen and mixed to prepare standards solution (**Figure 1**). They were used to optimize the 2D-LC conditions.

### Optimization of HILIC

**FIGURE 2:**
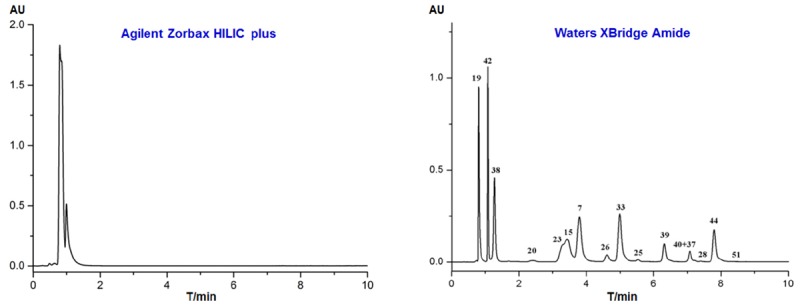
HPLC chromatograms of 16 reference standards separated with different HILIC columns with acetonitrile and 0.1% formic acid as the mobile phase (254 nm).

Two types of HILIC columns (Agilent Zorbax HILIC plus and Waters XBridge Amide) were evaluated by separating the above standard solution, with the gradient program described in Section “Off-Line Two-Dimensional Liquid Chromatography.” As shown in **Figure 2**, all the standards were co-eluted within 2 min with poor separation when they were separated on the Agilent Zorbax HILIC plus column. On the contrary, the Waters XBridge Amide column exhibited stronger retention capacity to the standards, and their identities were confirmed by mass spectrometry (**Figure 2**). A total of 14 peaks can be observed, and only four compounds were not fully separated. Therefore, we chose Amide as the stationary phase for the first dimension HILIC in this study, and gradient acetonitrile and 0.1% formic acid were chosen as the mobile phases.

### Optimization of RPLC

**FIGURE 3:**
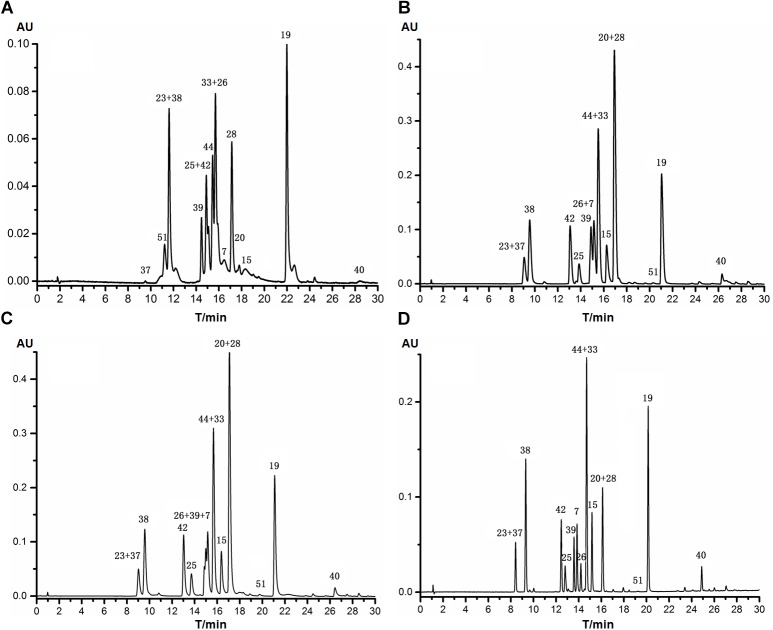
HPLC chromatograms of 16 reference standards separated with different Agilent RPLC columns with methanol and 0.1% formic acid as the mobile phase (254 nm). **(A)** Zorbax SB-C8 (4.6 mm × 150 mm, 5.0 μm); **(B)** Zorbax SB-C18 (2.1 mm × 100 mm, 3.5 μm); **(C)** Eclipse plus C18 (2.1 mm × 100 mm, 3.5 μm); **(D)** Eclipse plus C18 (4.6 mm × 100 mm, 3.5 μm).

Four RPLC columns from Agilent Technologies with different packing materials or inner diameters were tested in respect of selectivity and resolution by using the gradient program described in Section “Off-Line Two-dimensional Liquid Chromatography.” The 16 reference standards were difficult to be fully separated in one single run, as shown in **Figure 3**. Apparently, the Eclipse plus C18 (4.6 mm, i.d.) column showed the highest resolution and best peak shape, and only six analytes were not fully separated. In particular, compounds **7**, **26**, and **39** could be well-resolved, though they were almost overlapped when separated on Zorbax SB-C8, Zorbax SB-C18 and Eclipse plus C18 (2.1 mm, i.d.). Therefore, Agilent Eclipse plus C18 (4.6 mm, i.d.) was tentatively chosen as the stationary phase for the second dimension RPLC. The mobile phases were then optimized, and detailed experimental process was described in Supporting information. As a result, 0.1% formic acid was used as the aqueous phase, and methanol was used as the organic phase.

**FIGURE 4:**
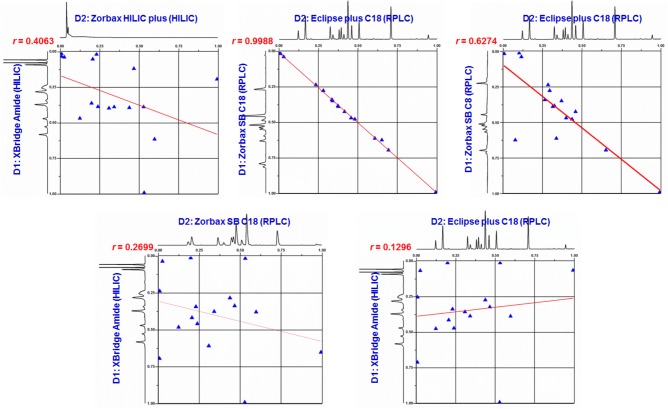
Normalized retention time plots for HILIC × HILIC, RPLC × RPLC, and HILIC × RPLC systems.

Subsequently, the orthogonality between Waters XBridge Amide and Agilent Eclipse plus C18 (HILIC × RPLC) was evaluated with Pearson linearity regression correlation coefficient (*r*) as the index. In addition, the orthogonality of several other combination modes, namely XBridge Amide × Zorbax HILIC plus, Zorbax SB C18 × Eclipse plus C18 (4.6 mm, i.d.), Zorbax SB C8 × Eclipse plus C18 (4.6 mm, i.d.) and XBridge Amide × Zorbax SB C18, was also determined. Retention times of each reference standard separated on these columns were firstly transformed into normalized retention times [${\mathrm{RT}}_{i(\mathrm{norm})}=\frac{{\mathrm{RT}}_{i}-{\mathrm{RT}}_{\min}}{{\mathrm{RT}}_{\max}-{\mathrm{RT}}_{\min}}$, calculated according to our previous publication (Ji et al., 2017)], and the *r*-values of the two groups of RT_(i)norm_ values were then calculated, respectively. As shown in **Figure 4**, the *r*-values of Amide × HILIC plus, SB C18 × plus C18 (4.6 mm, i.d.), SB C8 × plus C18 (4.6 mm, i.d.), Amide × SB C18 and Amide × plus C18 (4.6 mm, i.d.) were 0.4063, 0.9988, 0.6274, 0.2699, and 0.1296, respectively, suggesting that the combination modes of both HILIC × HILIC (Amide × HILIC plus) and RPLC × RPLC (SB C18 × plus C18 and SB C8 × plus C18) showed poor orthogonality with *r* > 0.40, due to the similar separation mechanisms between D1 and D2. As expected, the combination mode of HILIC × RPLC (Amide × SB C18 and Amide × plus C18) exhibited a higher orthogonality with *r* < 0.30. Then, the orthogonality $\left(O=\frac{\sum \mathrm{bins}-\sqrt{P_{\max}}}{0.63P_{\max}}\right)$ and practical peak capacity $\left(n_{2D}=n_{1}\times n_{2}\times \frac{1}{\beta }\times f\right)$ of different combination modes were calculated according to our previous publication (Ji et al., 2017). As a result (**Supplementary Table S1**), Amide × plus C18 gave a significantly higher practical peak capacity (2303 and 1231) than Amide × SB C18 (1691 and 850), calculated by two different methods, though their orthogonality was the same (0.3969). Based on the above data, the choice of Waters XBridge Amide × Agilent Eclipse plus C18 for the chemical analysis of SGT was further confirmed in this study.

### Evaluation of the Off-Line 2D-LC System

**FIGURE 5:**
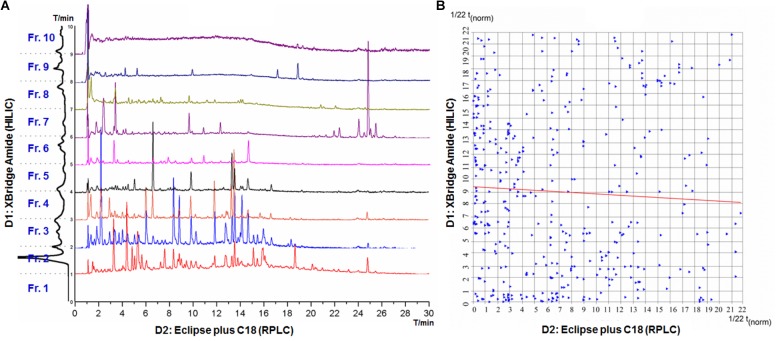
HPLC chromatograms and the 2D-LC plot of SGT. **(A)** HPLC chromatograms of nine fractions in the second dimension RPLC. **(B)** The normalized retention time plot for the chemical constituents of SGT separated by the 2D-LC system.

The optimized off-line 2D-LC system was used to analyze SGT, which mainly contains flavonoids, triterpenoid saponins, phenylethanoid glycosides, and organic acids. SGT was firstly separated on the XBridge Amide column in D1, and a total of 10 fractions (Fraction 1 to Fraction 10) were consecutively collected with 0.4 mL (1 min) per fraction (**Supplementary Figure S1**). The resulting fractions (except for Fraction 1) were further separated on the Zorbax Eclipse plus C18 column in D2, respectively, which was successively coupled with DAD and qTOF-MS as the detectors (**Figure 5A**). All the peaks in the nine chromatograms (Fraction 2 to Fraction 10) were assigned based on their retention time, UV spectra and MS data, and a total of 465 peaks were finally recognized in SGT. To evaluate the orthogonality and peak capacity of the 2D-LC system, the normalized retention times of all the peaks were calculated, and the separation space was divided into 22 × 22 rectangular bins, which (484) is close to P_max_ (465). The rectangular bins were then superimposed with the data points, as shown in **Figure 5B**, and orthogonality and peak capacity were calculated according to literatures (Gilar et al., 2005; Rutan et al., 2012). Bins containing data plots (Σbins) covered about 50% of the separation space, and the orthogonality was calculated as 63.62%. In addition, the Pearson correlation coefficient (*r*) of the two groups of normalized retention time values was 0.0525, which suggested a good orthogonality.

The average peak width of D1 and D2 was 0.738 and 0.145 min, respectively, and the effective gradient time for D1 and D2 was 10 and 30 min, respectively. Thus, the theoretical peak capacities for D1 and D2 were 14 and 207, respectively. The theoretical peak capacity of the 2D-LC system was 2898, and the practical peak capacity was 2399 and 1099, respectively, calculated following two methods described in our previous publication (Ji et al., 2017). The 2D-LC system increased the peak capacity by almost 11-fold comparing with the single-dimension D2. Improvement on peak capacity is beneficial to the exposure of more minor components, which could contribute to comprehensive separation and detection of chemical constituents in SGT and its component herbs.

### Separation and Characterization of Chemical Constituents in SGT and Its Component Herbs by the Off-Line 2D-LC/qTOF-MS System

Chemical constituents of SGT were globally analyzed by the optimized off-line 2D-LC/qTOF-MS system, and a total of 465 peaks were detected. The eight component herbs were also respectively separated and detected by using the 2D-LC system, and we found that 12 peaks detected in SGT were not discovered in any component herbs. Their retention times and high-resolution mass spectral data were listed in **Supplementary Table S2**. The 12 compounds might be produced through chemical reactions of chemical constituents in component herbs during decocting together, such as hydrolysis, oxidation, and dissolution. They might cause the pharmacological activity differences between SGT and its eight component herbs, and contribute to exploration of the herbal compatibility mechanism. Further separation and purification are necessary to fully identify the chemical structures of the 12 compounds through nuclear magnetic resonance (NMR) spectroscopic analysis in future study.

**FIGURE 6:**
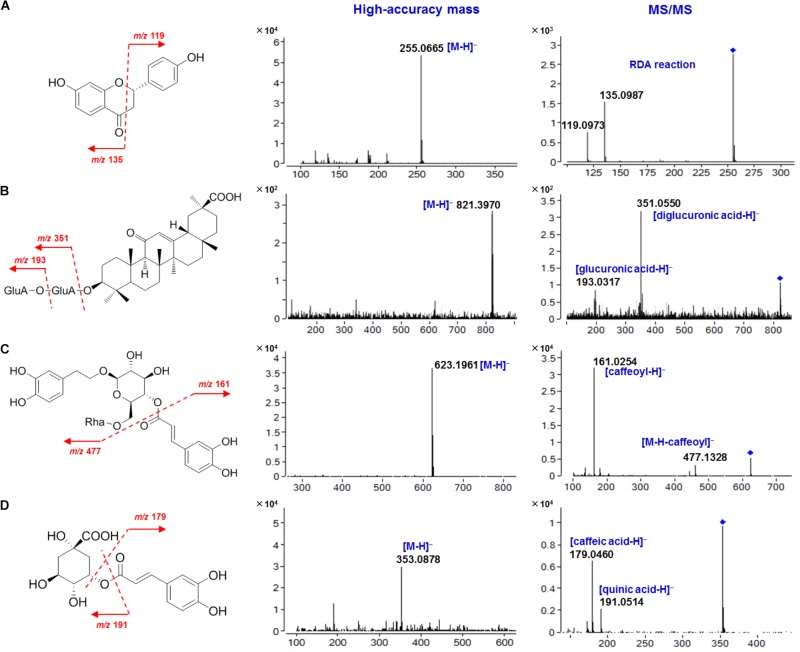
High-accuracy mass and MS/MS spectra of four representative compounds in SGT. **(A)** Liquiritigenin (**5**); **(B)** Glycyrrhizic acid (**40**); **(C)** Forsythoside A (**39**); **(D)** Chlorogenic acid (**23**).

Among the 465 peaks, a selected group of constituents (54 compounds) were identified by comparing with reference standards (23 compounds), or tentatively characterized by comparing their high-resolution mass spectral data with previous literatures (31 compounds). These compounds included free flavonoids, flavonoid glycosides, triterpene saponins, phenylethanoid glycosides, organic acids, and others (**Table 1**). The qTOF-MS spectra of four representative compounds are illustrated in **Figure 6**.

**Table 1:** Characterization of selected chemical constituents in SGT by qTOF-MS analysis.

No.	Fr.	*t*_R_ (min)	Formula	Predicted [M-H]^-^	Measured [M-H]^-^	Error (ppm)	Identification	Major fragment ions	Source
1	2	2.15	C_8_H_14_O_4_	173.0819	173.0824	2.9	Rengynic acid	155.0187, 130.9664	F
2	2	7.00	C_20_H_30_O_12_	461.1664	461.1665	0.2	Forsythoside E	315.1216, 108.0212	F
3^∗^	2	8.49	C_16_H_12_O_7_	315.0510	315.0529	6.0	Isorhmnetin	246.9526, 207.0309	M
4	2	9.19	C_29_H_36_O_16_	639.1931	639.1915	-2.5	Forsythoside C	621.2144, 487.1683	F
5	2	11.53	C_15_H_12_O_4_	255.0663	255.0665	0.8	Liquiritigenin	135.0987, 119.0973	L
6^∗^	2	11.99	C_16_H_12_O_6_	299.0561	299.0547	-4.7	Diosmetin	231.0859, 151.0047	C
7^∗^	2	12.08	C_25_H_24_O_12_	515.1195	515.1182	-2.5	Isochlorogenic acid A	353.1254, 179.1406	C, M
8	2	12.22	C_26_H_30_O_13_	549.1614	549.1610	-0.7	Licuraside	255.0691, 135.0090	L
9	2	12.35	C_21_H_22_O_9_	417.1191	417.1179	-2.9	Isoliquiritin	255.0681, 135.0085,	L
10^∗^	2	13.75	C_15_H_10_O_6_	285.0405	285.0392	-4.6	Kaempferol	238.9991, 185.0123	C, L
11	2	14.00	C_21_H_22_O_10_	433.1140	433.1124	-3.7	5-Hydroxy-liquiritin	271.0601, 151.0410,	L
12	2	14.09	C_23_H_24_O_10_	459.1297	459.1288	-2.0	Liquiritigenin-4′-*O*-(6-*O*-acetyl)-glucoside	255.0661, 134.9757	L
13	2	14.21	C_16_H_18_O_9_	353.0878	353.0885	2.0	1-Caffeoyl-quininic acid	191.0684, 161.0344	M
14^∗^	2	14.30	C_15_H_10_O_7_	301.0354	301.0325	-9.6	Quercetin	178.9282, 151.0496	F, C, L
15^∗^	2	14.64	C_25_H_24_O_12_	515.1195	515.1207	2.3	Isochlorogenic acid C	353.0944, 191.0433	C, M
16	2	15.38	C_26_H_30_O_13_	549.1614	549.1639	4.5	Isoliquiritin apioside	417.1165, 255.0654	L
17	2	15.60	C_21_H_22_O_9_	417.1191	417.1178	-3.1	Neoliquiritin	255.0657, 135.0088	L
18	2	15.90	C_21_H_22_O_9_	417.1191	417.1199	1.9	Neoisoliquiritin	255.0648, 135.0083	L
19^∗^	2	16.08	C_15_H_10_O_5_	269.0455	269.0443	-4.5	Apigenin	151.0977, 117.1972	C
20^∗^	2	17.09	C_27_H_34_O_11_	533.2028	533.2019	-1.7	Forsythin	371.0965	F
21	2	20.04	C_44_H_64_O_19_	895.3969	895.3952	-1.9	22β-Acetoxyl-licorice-saponin G2	832.9889, 351.0551	L
22	3	1.91	C_7_H_12_O_6_	191.0561	191.0568	3.7	Quinic acid	173.0419, 110.0973	S, L, C, F
23^∗^	3	8.00	C_16_H_18_O_9_	353.0878	353.0878	0.0	Chlorogenic acid	191.0514, 179.0460	C, M
24	3	10.54	C_27_H_32_O_13_	563.1770	563.1798	5.0	Isoliquiritoside	473.0765, 327.0865	L
25^∗^	3	11.46	C_21_H_22_O_9_	417.1191	417.1203	2.9	Liquiritin	255.0701, 134.9676	L
26^∗^	3	13.75	C_21_H_20_O_11_	447.0933	447.0974	9.2	Luteolin-7-*O*-glucoside	285.1876, 175.1059	C
27	3	15.05	C_27_H_30_O_13_	561.1614	561.1605	-1.6	Glycyroside	267.0653, 146.9658	L
28^∗^	3	15.43	C_21_H_20_O_11_	447.0933	447.0936	0.7	Astragalin	285.0438, 151.0036	M, L, F
29^∗^	3	18.28	C_15_H_10_O_6_	285.0405	285.0401	-1.4	Luteolin	151.0029, 133.0308	C
30	4	11.19	C_29_H_36_O_15_	623.1981	623.1968	-2.1	Forsythoside I	477.1656, 315.1085	F
31^∗^	4	12.10	C_28_H_32_O_14_	591.1719	591.1711	-1.3	Buddleoside	282.9350, 238.9306	C
32	4	12.82	C_29_H_36_O_15_	623.1981	623.1979	-0.3	Forsythoside H	477.0351, 161.0244	F
33^∗^	4	14.09	C_21_H_20_O_12_	463.0882	463.0877	-1.1	Quercetin-3-*O*-glucoside	300.0363, 151.0102	C, M
34	4	20.58	C_42_H_60_O_16_	819.3809	819.3757	-6.3	Licorice-saponin E2	757.3807, 643.3443	L
35	4	21.17	C_44_H_64_O_18_	879.4020	879.3969	-5.8	22β-Acetoxyl-glycyrrhizic acid	703.1909, 350.9911	L
36	4	21.58	C_42_H_62_O_17_	837.3914	837.3924	1.2	Licorice-saponin G2	661.3565, 485.2294	L
37^∗^	5	7.74	C_20_H_27_NO_11_	456.1506	456.1501	-1.1	Amygdalin	294.0426, 131.1087	S
38^∗^	5	9.08	C_9_H_8_O_4_	179.0350	179.0359	5.0	Caffeic acid	161.1098, 135.0446	R, F, C, S
39^∗^	5	12.07	C_29_H_36_O_15_	623.1981	623.1961	-3.2	Forsythoside A	477.1328, 161.0254	F
40^∗^	5	24.16	C_42_H_62_O_16_	821.3965	821.3970	0.6	Glycyrrhizic acid	351.0550, 193.0317	L
41	6	3.81	C_14_H_24_O_9_	335.1348	335.1326	-6.6	Rengynic acid-1′-*O*-glucoside	173.0578	F
42^∗^	6	4.22	C_10_H_10_O_4_	193.0506	193.0502	-2.1	Ferulic acid	149.0605, 134.0354	C, R
43	6	24.16	C_42_H_62_O_16_	821.3965	821.3967	0.2	Uralsaponin A	645.1405, 469.1888	L
44^∗^	7	11.93	C_27_H_30_O_16_	609.1461	609.1449	-2.0	Rutin	301.0498, 285.1097	C, M, F
45	7	13.61	C_25_H_24_O_12_	515.1195	515.1154	-7.9	Isochlorogenic acid B	173.0481, 161.0267	C
46	7	20.19	C_59_H_94_O_29_	1265.5808	1265.5902	7.4	Platycodin A	1133.512, 681.4071	P
47	7	23.91	C_48_H_72_O_21_	983.4493	983.4523	3.0	Licorice-saponin A3	645.1236, 351.0817	L
48	7	24.15	C_42_H_62_O_16_	821.3965	821.3930	-4.3	Licorice-saponin H2	469.3447, 351.0641	L
49	7	24.83	C_42_H_62_O_16_	821.3965	821.3973	1.0	Licorice-saponin K2	645.2346, 351.0054	L
50^∗^	8	3.66	C_21_H_20_O_12_	463.0882	463.0902	4.3	Hyperoside	300.9523	F
51^∗^	8	19.77	C_57_H_92_O_28_	1223.5702	1223.5699	-0.2	Platycodin D	959.4888, 681.3826	P
52	8	20.15	C_57_H_92_O_27_	1207.5753	1207.5690	-5.2	Polygalacin D	1075.4732, 665.3538	P
53	8	20.23	C_58_H_94_O_28_	1237.5859	1237.5841	-1.4	Platycoside A	1027.1965, 695.1736	P
54	9	16.60	C_69_H_112_O_38_	1547.6759	1547.6749	-0.6	Platycoside E	1415.6212, 989.0768	P


*^∗^Identified by comparing with reference standards.*

*Fr., fractions collected from the first dimension HILIC; M, mulberry leaf; C, chrysanthemum; F, Fructus *Forsythiae*; L, licorice; S, Semen *Armeniacae* Amarum; P, *Platycodi* Radix; R, *Phragmitis* Rhizoma.*

### Characterization of Flavonoids

Flavonoids (free flavonoids and flavonoid glycosides) in SGT are mainly derived from mulberry leaf, chrysanthemum, Fructus *Forsythiae* and licorice. In the negative ion mode, free flavonoids in SGT could fragment on ring-C following the retro Diels-Alder (RDA) reaction at a relatively high collision energy, and they could also lose small molecules (like CO) or radicals (like CH_3_⋅) to produce diagnostic fragments (Fabre et al., 2001). For the flavonoid glycosides in SGT, flavonoid *O*-glycosides could undergo collision-induced dissociation (CID), and produce [M-H-162]^-^ (-glucose), [M-H-146]^-^ (-rhamnose) or [M-H-132]^-^ (-apiose) ions by the neutral loss of sugar moieties. The resulting aglycones further fragment to produce the same fragments as the corresponding free flavonoids (Cuyckens et al., 2001). In contrast, flavonoid *C*-glycosides could fragment on the sugar ring to lose 60, 90, or 120 Da (^0,4^X, ^0,3^X, or ^0,2^X cleavage, respectively) at a relatively high collision energy (Lin et al., 2005). Based on these fragmentation behaviors, a selected group of flavonoids (7 free flavonoids and 16 flavonoid glycosides), including isoflavones, flavones, flavanones and chalcones, were identified or tentatively characterized in SGT. Here we choose liquiritigenin and its analogs, as well as their glycosides, as examples to describe their structural characterization.

Liquiritigenin (**5**) is the main free flavanone in licorice. Its [M-H]^-^ ion at *m/z* 255.0665 fragmented to give *m/z* 135.0987 and *m/z* 119.0973 following the RDA reaction (Qiao et al., 2015). Liquiritin (liquiritigenin-4′-*O*-glucoside, **25**) is a liquiritigenin *O*-glucoside with a [M-H]^-^ ion at *m/z* 417.1203, and it eliminated a glucosyl residue to produce *m/z* 255.0701 corresponding to the aglycone. Like liquiritigenin, this fragment further fragmented to give *m/z* 134.9676 following the RDA reaction. Compound **9** (isoliquiritin) is a chalcone *O*-glucoside, and it showed the same fragmentation behavior as **25**. However, its retention time on the Agilent Eclipse Plus C18 column was shorter than **25** (Qiao et al., 2015). Compound **11** (5-hydroxy-liquiritin) is also a flavanone *O*-glucoside, and its [M-H]^-^ ion at *m/z* 433.1124 could easily lose a glucosyl residue to produce *m/z* 271.0601, which further fragmented to give *m/z* 151.0410 following the RDA reaction. This indicated that **11** was a hydroxylated derivative of **25** (Montero et al., 2016). Similarly, compounds **8**, **12**, **16, 17, 18**, **24,** and **27** were tentatively assigned to licuraside, liquiritigenin-4′-*O*-(6-*O*-acetyl)-glucoside, isoliquiritin apioside, neoliquiritin, neoisoliquiritin, isoliquiritoside, and glycyroside, respectively (Zhou et al., 2004; Qiao et al., 2015; Montero et al., 2016). The other selected flavonoids (**3**, **6**, **10**, **14**, **19**, **26**, **28**, **29**, **31**, **33**, **44,** and **50**) were identified by comparing with reference standards.

### Characterization of Triterpene Saponins

Triterpene saponins in SGT are mainly derived from *Platycodi* Radix and licorice, and most of them are oleanane-type triterpenes, which contain triterpene oleanolic acid or its analogs as the sapogenin and one or two oligosaccharide chains substituted at 3-OH or 28-OH. In the negative ion mode, they could lose sugar residues or sapogenins to produce corresponding diagnostic fragments. Based on this, the structures of 14 selected triterpene saponins were identified or tentatively characterized in this study.

Glycyrrhizic acid (**40**), with two connected glucuronic acid units as the saccharide chain, generated an [M-H]^-^ ion at *m/z* 821.3970, indicating a molecular formula of C_42_H_62_O_16_. The [M-H]^-^ ion fragmented into two characteristic fragments at *m/z* 351.0550 ([diglucuronic acid-H]^-^) and *m/z* 193.0317 ([glucuronic acid-H]^-^), respectively. Structural analogs of **40**, including 22β-acetoxyl-licorice-saponin G2 (**21**), licorice saponin E2 (**34**), 22β-acetoxyl-glycyrrhizic acid (**35**), licorice-saponin G2 (**36**), uralsaponin A (**43**), licorice-saponin A3 (**47**), licorice-saponin H2 (**48**) and licorice-saponin K2 (**49**), were tentatively characterized (Zhou et al., 2004; Qiao et al., 2015; Montero et al., 2016). Platycodin D (**51**) contains two oligosaccharide chains, which are composed of five sugar residues, namely glucose, arabinose, rhamnose, xylose, and apiose. It had the molecular formula of C_57_H_92_O_28_ based on its [M-H]^-^ ion at *m/z* 1223.5699, and its characteristic fragments were *m/z* 959.4888 ([M-apiose-xylose-H]^-^) and *m/z* 681.3826 ([*M*-apiose-xylose-rhamnose-arabinose-H]^-^), respectively. According to the similar fragmentation behaviors, the other selected saponins from *Platycodi* Radix (**46**, **52, 53,** and **54**) were tentatively characterized (Ha et al., 2010; Lee et al., 2015).

### Characterization of Phenylethanoid Glycosides

Five selected phenylethanoid glycosides characterized in SGT (**2**, **4**, **30**, **32,** and **39**) were mainly derived from Fructus *Forsythiae*, and their chemical structures contained one or more aromatic rings, glucoses and caffeic acids. The [M-H]^-^ ion of forsythoside A (**39**) at *m/z* 623.1961 suggested a molecular formula of C_29_H_36_O_15_, and the fragments at *m/z* 477.1328 and *m/z* 161.0254 were attributed to the loss of a caffeoyl group ([M-H-162]^-^) and the caffeoyl group ([caffeoyl-H]^-^), respectively. Similarly, forsythoside E (**2**), forsythoside C (**4**), forsythoside I (**30**), and forsythoside H (**32**) were tentatively characterized (Zhang et al., 2016).

### Characterization of Phenolic Acids

Almost all of the component herbs contain phenolic acids, and a total of 10 selected phenolic acids in SGT were identified or tentatively characterized in this study. In the negative ion mode, phenolic acids could lose a carboxy group with the neutral loss of 44 Da (CO_2_) to generate diagnostic fragments, and the loss of small molecules (like H_2_O) or radicals (like CH_3_⋅) sometimes also occurred. For example, the [M-H]^-^ ion of ferulic acid (**42**) at *m/z* 193.0502 produced characteristic fragments at *m/z* 149.0605 ([M-H-CO_2_]^-^) and *m/z* 134.0354 ([M-H-CH_3_⋅]^-^). For chlorogenic acid (**23**), where caffeic acid forms an ester bond with quinic acid, the ester bond fragmented to produce *m/z* 191.0514 and *m/z* 179.0460, representing a quinic acid unit and a caffeic acid unit, respectively. Based on these fragmentation patterns, the other selected phenolic acids (**1**, **7**, **13**, **15**, **22**, **38**, **41,** and **45**) were tentatively characterized (Wang et al., 2014; Yan et al., 2014; Lee et al., 2015; Zhang et al., 2016; Zhu et al., 2017).

## Conclusion

In summary, we developed an off-line HILIC × RP 2D-LC system to comprehensively separate the chemical constituents in SGT and its component herbs. The 2D-LC system showed high orthogonality (63.62%) and approximate 11-fold improvement in peak capacity in contrast to conventional one-dimensional RPLC separation. As a result, a total of 465 peaks were detected, and the structures of 54 selected compounds were fully identified or tentatively characterized by qTOF-MS analysis. In addition, 12 peaks detected in SGT were not discovered in any component herbs, and they might contribute partly to exploration of the compatibility mechanism of the component herbs. Integration of off-line HILIC × RP 2D-LC and high-resolution mass spectrometry is proven as a promising tool for chemical profiling and comparison of complicated Chinese patent medicines and their component herbs.

## Author Contributions

SJ, M-ZG, and D-QT: participated in research design. SJ, Z-ZL, YD, and Z-YS: performed the experiments and data analysis. JW, T-YW, JH, and D-ZY: contributed to the writing of the manuscript.

## Conflict of Interest Statement

The authors declare that the research was conducted in the absence of any commercial or financial relationships that could be construed as a potential conflict of interest.
