# Supplementary material for: Chemical Profiling and Comparison of Sangju Ganmao Tablet and Its Component Herbs Using Two-Dimensional Liquid Chromatography to Explore Compatibility Mechanism of Herbs
Source: Front Pharmacol. 2018 Oct 16;9:1167. doi: 10.3389/fphar.2018.01167 (PMC6198175; doi:10.3389/fphar.2018.01167)
Supplement: Supplementary file 1 [file Table_1.DOCX]

**Chemical profiling and comparison of Sangju Ganmao tablet and its component herbs using two-dimensional liquid chromatography to explore compatibility mechanism of herbs**

Shuai Ji ^a,c,#^, Zhan-zhong Liu ^b,#^, Jing Wu ^d^, Yan Du ^a^, Zhen-yu Su ^c^, Tian-yun Wang ^c^, Jie Han ^c^, Dong-zhi Yang ^a,c^, Meng-zhe Guo ^a,c^, Dao-quan Tang ^a,c,*^

^a^ *Department of Pharmaceutical Analysis, Xuzhou Medical University, Xuzhou 221004, China*

^b^ *Department of pharmacy, Xuzhou Infectious Disease Hospital, Xuzhou 221004, China*

^c^ *Jiangsu Key Laboratory of New Drug Research and Clinical Pharmacy, Xuzhou Medical University,* *Xuzhou 221004, China*

^d^ *Department of Pharmaceutical Analysis, Jiangsu College of Nursing, Huai’an 223001, China*

^#^ The first two authors (S. Ji and Z. Liu) contributed equally to this paper.

*Corresponding author. Tel.: +86 516 83263133. Fax: +86 516 83263133. E-mail address: tangdq@xzhmu.edu.cn or tdq993@hotmail.com.

**Optimization of mobile phases used in the second dimension RPLC (Eclipse plus C18)**

The aqueous phase and organic phase were respectively optimized in this study. For the aqueous phase, the addition of 0.1% formic acid/10 mM ammonium formate and 0.1% acetic acid/10 mM ammonium acetate were respectively tested, and the former showed a better separation for the 16 reference standards. Next, the addition of different concentration of formic acid/ammonium formate, namely 0.05% formic acid/5 mM ammonium formate, 0.1% formic acid/10 mM ammonium formate and 0.2% formic acid/15 mM ammonium formate, were compared, and water containing 0.1% formic acid/10 mM ammonium formate gave the highest resolution. Moreover, we found that absence or presence of ammonium formate in water hardly affected the HPLC chromatograms, and thus, water containing 0.1% formic acid was used as the aqueous phase. For the organic phase, methanol and acetonitrile were compared, and the results showed that better separation can be obtained when methanol was used.

**Preparation of the simulative solution of Sangju Ganmao tablet (SGT).**

The simulative solution of SGT was prepared according to its record in China Pharmacopoeia [3]. Briefly, Fructus *Forsythiae* (0.6 g) was firstly extracted through steam distillation to obtain the volatile oil, and the resulting residue was decocted in water together with mulberry leaf (0.75 g), chrysanthemum (0.3 g), licorice (0.24 g), Semen *Armeniacae* Amarum (0.6 g) and *Phragmitis* Rhizoma (0.45 g) for twice (2 h each time)*.* After concentration in vacuum, *Platycodi* Radix (0.6 g), the volatile oil from Fructus *Forsythiae* and mint (1.6 µL) were added into the extract, and they were ultrasonically extracted in 50 mL of 50% methanol twice (45 min for the first time and 30 min for the second time). The decoctions were filtered to remove the herbal residue, combined, and concentrated in vacuum at 40 °C. The resulting residue was reconstituted in 10 mL of 50% methanol, and the samples were filtered through 0.22 μm membranes before use. The solutions of the eight component herbs were respectively prepared using the same method as described above for the simulative solution of SGT.

**Table S1**. Orthogonality (*O*) and practical peak capacity (*n*_2D_) values of off-line 2D-LC systems with different combination modes by separating 16 reference standards in SGT. ^a^

| Combination modes | *n*_1_ | *n*_2_ | t_s_ (min) | σ_1_ (min) | 1/β | *r* | *O* | *f* ^b^ | *f* ^c^ | *n*_2D_^b^ | *n*_2D_^c^ |
| --- | --- | --- | --- | --- | --- | --- | --- | --- | --- | --- | --- |
| A | 14 | 23 | 1 | 0.738 | 0.8495 | 0.4063 | 0.3969 | 0.7876 | 0.5000 | 215 | 137 |
| B | 143 | 207 | 1 | 0.210 | 0.4166 | 0.9988 | 0.0000 | 0.0477 | 0.2500 | 588 | 3083 |
| C | 120 | 207 | 1 | 0.251 | 0.4789 | 0.6274 | 0.2976 | 0.6474 | 0.4375 | 7701 | 5204 |
| D | 14 | 143 | 1 | 0.738 | 0.8495 | 0.0109 | 0.3969 | 0.9945 | 0.5000 | 1691 | 850 |
| E | 14 | 207 | 1 | 0.738 | 0.8495 | 0.1296 | 0.3969 | 0.9354 | 0.5000 | 2303 | 1231 |

A, XBridge Amide × Zorbax HILIC plus; B, Zorbax SB C18 × Eclipse plus C18; C, Zorbax SB C8 × Eclipse plus C18; D, XBridge Amide × Zorbax SB C18; E, XBridge Amide × Eclipse plus C18.

^a^ The orthogonality (*O*) and practical peak capacity (*n*_2D_) of different combination modes were calculated according on our previous publication (Ji et al., 2017).

^b^ Calculated according to Carr et al. (Gilar et al., 2005).

c Calculated according to Gilar et al. (Rutan et al., 2012).

**Table S2**. Retention time and high-resolution mass spectral data of 12 peaks only detected in SGT.

| **No.** | Fraction | *t*_R_ (min) | Identification | Measured [M-H]^−^ | Major fragment ions |
| --- | --- | --- | --- | --- | --- |
| **a** | 1 | 3.58 | Unknown | 171.0217 | 130.9662 |
| **b** | 1 | 3.94 | Unknown | 122.0370 | 108.0215 |
| **c** | 1 | 21.19 | Unknown | 248.9632 | 204.9721, 154.9741, 136.9839 |
| **d** | 2 | 11.07 | Unknown | 359.0854 | 312.1138, 243.0663, 150.0568 |
| **e** | 5 | 19.53 | Unknown | 1265.6198 | 1091.5502, 733.5471, 421.3178, 113.0241 |
| **f** | 6 | 19.36 | Unknown | 1133.5715 | 681.4031, 501.3312, 337.1200, 131.0350 |
| **g** | 8 | 1.79 | Unknown | 659.9858 | 569.8020, 368.9800, 127.9903 |
| **h** | 9 | 1.82 | Unknown | 1475.0006 | 1354.9932, 1150.9942, 989.0009, 826.9966 |
| **i** | 10 | 0.97 | Unknown | 695.2443 | 649.2388, 517.1913, 191.0597 |
| **j** | 10 | 1.15 | Unknown | 845.1036 | 683.0707, 503.0379, 341.0121 |
| **k** | 10 | 1.75 | Unknown | 594.1718 | 564.1599, 503.1759, 386.1032, 179.0565 |
| **l** | 10 | 16.45 | Unknown | 1547.7215 | 1387.3977, 1136.5966, 969.3287, 643.2475 |

**Fig. S1**. The HPLC chromatogram of SGT separated by the XBridge Amide column at 254 nm.
